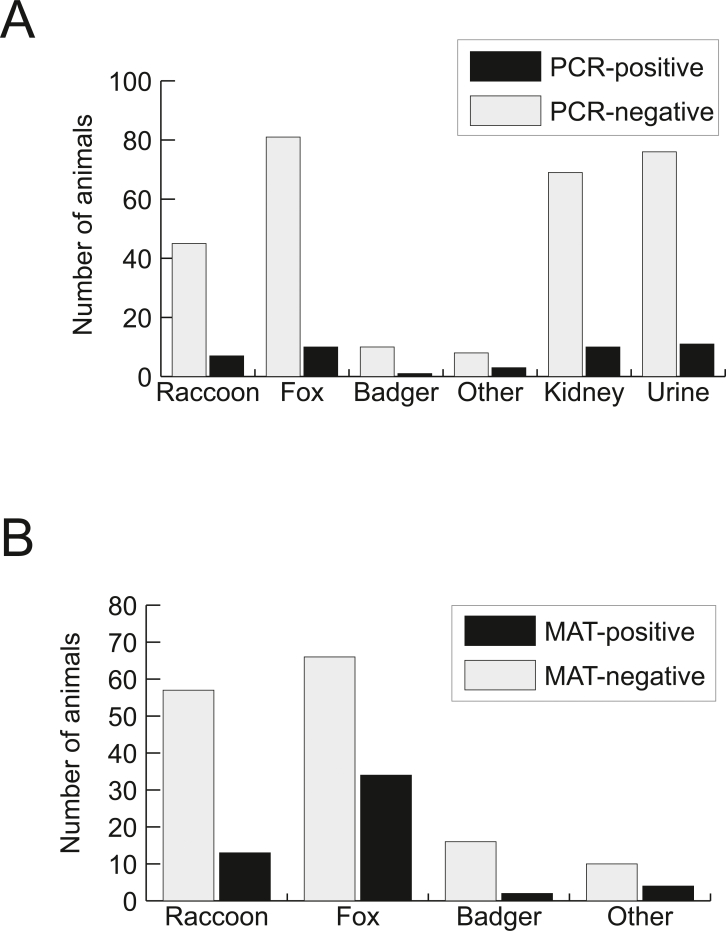# Serological and molecular detection as well as typing of *Leptospira* spp. in foxes, raccoons, and other wild carnivores in North-Eastern Germany, 2021–2022

**DOI:** 10.1016/j.heliyon.2023.e23268

**Published:** 2023-12-03

**Authors:** Peter Kuhnert, Isabelle Brodard, Stefanie Ackermann, Peter Schierack, Joerg Jores

**Affiliations:** a Institute of Veterinary Bacteriology, Vetsuisse Faculty, University of Bern, Bern, Switzerland; b Institute of Biotechnology, Faculty Environment and Natural Sciences, Brandenburg University of Technology, Cottbus-Senftenberg, Senftenberg, Germany; c Faculty of Health Sciences Brandenburg, Brandenburg University of Technology Cottbus-Senftenberg, Senftenberg, Germany; d Multidisciplinary Center for Infectious Diseases, University of Bern, Bern, Switzerland

**Keywords:** Zoonosis, One health, MLST, Wildlife, *Brucella*, *Francisella tularensis*

## Abstract

Leptospirosis is a worldwide zoonosis caused by pathogenic *Leptospira* spp. While the latter are reported from various mammal hosts such as humans, dogs, or rodents, less is known about their presence in wild carnivores. We therefore investigated the presence of *Leptospira* spp. in foxes, raccoons, badgers, raccoon dogs, and martens in North-Eastern Germany. Kidney, urine, and blood specimens obtained from legally hunted or road-killed animals were tested by real-time PCR and by serogroup specific antibody detection for the presence of *Leptospira* spp. Additionally, kidney and urine specimens were tested by real-time PCR for the presence of *Brucella* spp. and *Francisella tularensis*, with all being negative for these two zoonotic pathogens. *Leptospira* spp. were detected by PCR in 12.6 % (n = 21/166) and serologically in 26.2 % (n = 53/202) of tissue and serum samples, respectively. Antibodies to 15 different serogroups were identified with Javanica (n = 25) and Bataviae (n = 12) being predominant. A high sero-prevalence of 34.0 % and 18.6 % in foxes and raccoons, respectively, and the presence of ST17 associated with human and animal leptospirosis indicates a reservoir and the zoonotic potential of these wild animals.

## 1 Introduction

Leptospirosis is a worldwide observed zoonotic disease caused by pathogenic *Leptospira* spp. In Germany the incidence of human leptospirosis is highest in regions in the North and South [1]. Transmission may occur through direct or indirect exposure to infected animals that carry the pathogen in their renal tubules and shed the *Leptospira* spp. in their urine. Canine and human leptospirosis are most prominent, but it can also affect other mammal species. Moreover, recent studies also showed the presence of *Leptospira* spp. in wild animals such as rodents, rabbits, wild boar, wolves, as well as zoo animals [[2], [3], [4], [5]]. Wild carnivores such as foxes, raccoons, badgers, raccoon dogs and martens regularly approach farmland as well as urban and peri-urban settlements, especially as a consequence of their diminishing natural habitats. Urban foxes, raccoons, and stone martens have been reported in Germany and elsewhere. They can shed infectious pathogens and at the same time pick up pathogens in the area they inhabit or visit. In particular, the raccoon is not native to Germany and its release into the wild in the last century caused a massive size increase in population (estimated >1 Mill) and territorial coverage [6,7]. Therefore, it is safe to assume that parasites and microorganisms associated with these animals have spread over time as well. Since our knowledge of such pathogens and possible reservoir hosts is rather limited a “One Health” approach should include different actors from the ecosystem and consequently more data on such pathogens in predators would be helpful to evaluate and mitigate epidemiological risks. For this purpose, we investigated blood and tissue samples mainly from red fox (*Vulpes vulpes*) and raccoon (*Procyon lotor*) but also from badger (*Meles meles*), raccoon dog (*Nyctereutes procyonides*), pine marten (*Martes martes*) and stone marten (*Martes foina*) both serologically by serogroup specific antibody detection and genetically by real-time PCR for the presence of *Leptospira* spp. In parallel, tissue samples were also tested by real-time PCR for *Brucella* spp. and *Francisella tularensis*, two additional zoonotic pathogens found in wildlife [8,9].

## 2 Materials and methods

**Fig. 1:**
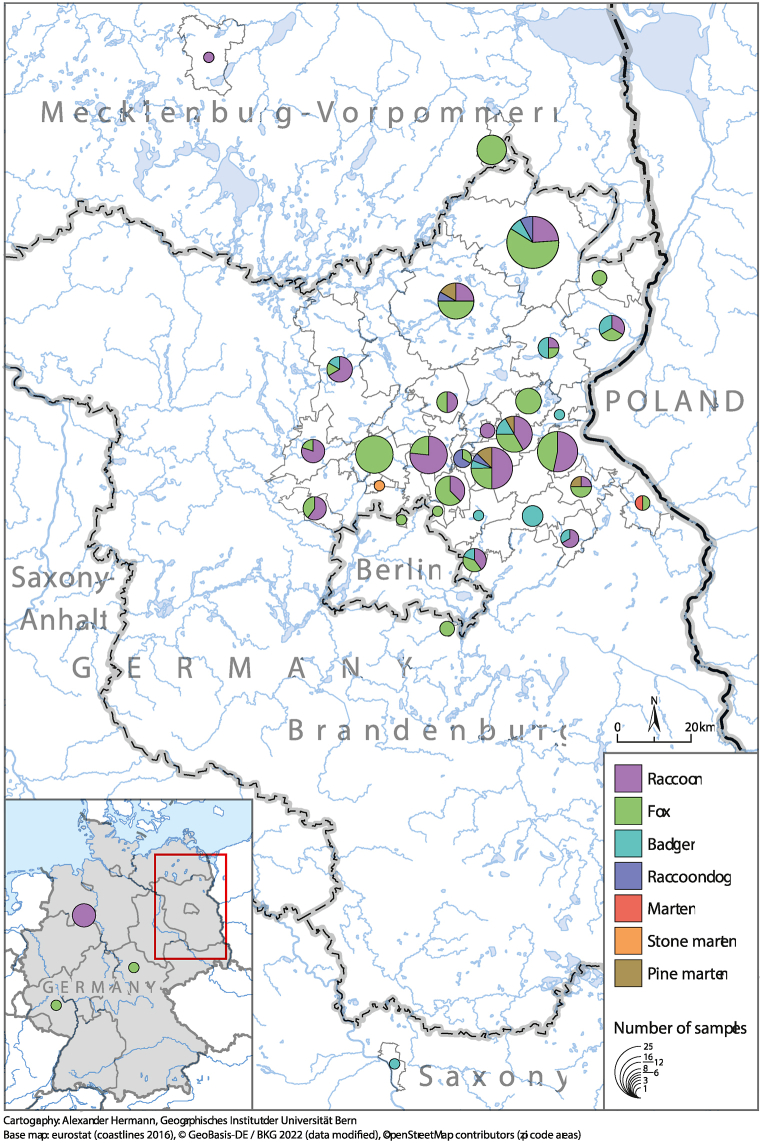
Map of sample origin. The main sampling region is zoomed out from the map of Germany. Samples from different wild carnivore species are indicated by color. The size of the circle indicates the number of samples. (For interpretation of the references to color in this figure legend, the reader is referred to the Web version of this article.)

A total of 202 serum samples were obtained from legally hunted or road-killed animals mainly in North-Eastern Germany in 2021–2022 (Fig. 1 and Table S1). From most of these animals (n = 166), kidney (K; n = 79) or urine (U; n = 87) specimens were also recovered with 91 red foxes (29 K/62 U), 52 raccoons (42 K/10 U), 11 badgers (1 K/10 U), 6 pine martens (3 K/3 U) as well as one stone marten (1 K), one marten not identified to this species level (1 K) and four raccoon dogs (2 K/2 U).

Specimens were collected by taxidermists, who received the animals mainly from hunters in the state of Brandenburg and stored the specimens at −20 °C. Subsequently, specimens were shipped to the laboratory and stored at −70 °C until processing. Blood samples were centrifuged and the serum in the supernatant collected. Total DNA isolation from kidney tissues and urine specimens was done using a semi-automatic extraction robot (King Fisher Duo Prime, Thermo Fisher Scientific, Reinach, Switzerland). The VetMAX™ Xeno™ Internal Positive Control (IPC) DNA (20,000 copies; Thermo Fisher Scientific) was added to each extraction sample before purification as a control. Purified DNA was stored at −20 °C until further analysis.

Real-time PCR for detection of *Leptospira* spp. based on the *lipL*32 gene was done as previously described but additionally extended by the IPC - VIC™ assay (Thermo Fisher Scientific) as an internal positive extraction and PCR amplification control [10,11]. Thereby PCR was performed in a total volume of 25 μL, 1× final concentration of TaqMan™ Fast Advanced PCR Master Mix, 1 μM of each primer, 80 nM of the probe, 1 μL IPC - VIC Primer Probe Mix, and 2.5 μL of the template. The following conditions were applied: 94 °C for 2 min, 45 cycles of 94 °C for 15 s, and 60 °C for 30 s using the TaqMan 7500 Fast Real-time PCR System. Samples were considered positive when showing an exponential amplification curve up to cycle 40 in both replicates.

Real-time PCR for *Brucella* spp. based on the IS*711* element was carried out according to Hinić et al. [12]. The real-time PCR for *F. tularensis* is based on the *fopA* gene and was done as described by Wicki et al. [13].

Multi-locus sequence typing (MLST) applying the scheme of Boonsilp et al. [14] was undertaken from samples being positive in the *Leptospira* spp. real-time PCR. PCR products confirmed by gel electrophoresis were purified using High Pure PCR Product Purification Kit (Roche Diagnostics GmbH, Mannheim, Germany) and sent for bi-directional Sanger sequencing (Microsynth, Balgach, Switzerland). Assembled and trimmed sequences were used for online allele and sequence type (ST) determination (https://pubmlst.org/leptospira) [15]. A minimum spanning tree (MST) was built using Bionumerics 8.1 based on the STs found in this study combined with corresponding ST entries from the pubMLST database (pubmlst.org/organisms/leptospira-spp).

Serology was performed using the microscopic agglutination test (MAT) [16] covering the following 19 *Leptospira* serogroups: Grippotyphosa, Australis, Pomona, Tarassovi, Canicola, Icterohaemorrhagiae, Sejroe, Bataviae, Autumnalis, Pyrogenes, Ballum, Andaman, Celledoni, Cynopteri, Hebdomadis, Javanica, Panama, Shermani, Semaranga.

The Wilson score interval was used for calculating the 95 % confidence interval (CI) (https://www.statskingdom.com/proportion-confidence-interval-calculator.html).

## 3 Results

**Fig. 2:**
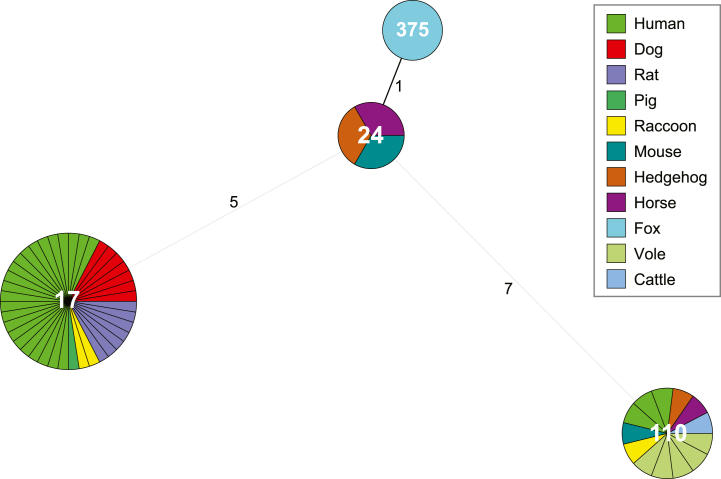
Minimum spanning tree (MST) and host association of sequence types (ST). The ST found in this study were combined with corresponding ST entries from the pubMLST database (pubmlst.org/organisms/leptospira-spp) with information on host in a MST analysis using Bionumerics 8.1. The fox and the three raccoon samples were determined in this study. The new ST375 found in fox is closely related to ST24 as indicated by the branch length and a single allele difference.

A total of 21 (12.6 %, 95 % CI: 8.4–18.6) of all 166 kidney or urine samples were positive for *Leptospira* spp. in the real-time PCR; ten from fox (11 %, 95 % CI: 6.1–19.1), seven from raccoon (13.5 %, 95 % CI: 6.7–25.3), one from badger (9 %, 95 % CI: 1.6–37.7), two from raccoon dog (50.0 %, 95 % CI: 15.0–85.0) and one from pine marten (16.7 %, 95 % CI: 3.0–56.4) (Fig. S1 Panel A). The samples from the stone marten and the marten were negative. There was no significant difference between proportions of positive samples in kidney or urine (12.7 %, 95 % CI: 7.0–21.8 versus 12.6 %, 95 % CI: 7.2–21.2). Amplification of MLST targets was only possible from samples showing Ct-values <34, however, only a fraction of those samples revealed all seven gene fragments (Table S1). Unambiguous genotype determination was possible with three raccoon samples with two of them harboring ST17 and one ST110. A new sequence type assigned ST375 was identified in one fox sample. In one raccoon sample the *tpiA* fragment could not be amplified but the remaining 6 alleles indicated ST17 as well. In four cases (three foxes and one raccoon dog) the genotype could be narrowed down to ST145, ST146, or ST335. In one pine marten the partial profile indicated a genotype related to ST117. A MST of the detected STs from this study and related STs from different hosts as deposited in the *Leptospira* spp. pubMLST database (pubmlst.org/organisms/leptospira-spp) is shown in Fig. 2. Thereby ST17 is most often found in humans but also in dogs and rats. Moreover, the new ST375 found in one fox is closely related to ST24 (only one base pair difference in *glmU*).

**Fig. 3:**
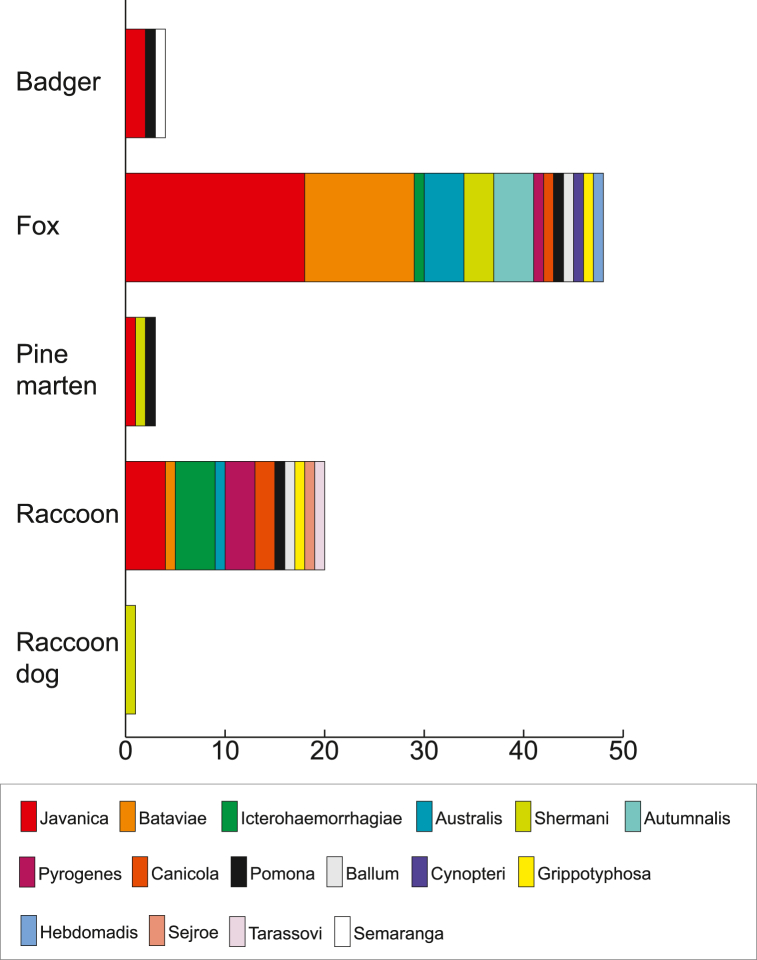
Distribution of serogroups detected in badger, fox, pine marten, raccoon, and raccoon dog. The number of samples reacting with the specific serogroup is indicated.

A total of 53 (26.2 %, 95 % CI: 20.7–32.7) of the 202 serum samples tested positive in the MAT. Sero-prevalence was found to be highest in foxes (34.0 %, 95 % CI: 25.5–43.7) followed by raccoons (18.6 %, 95 % CI: 11.2–29.2) and badgers (11.1 %, 95 % CI:3.1–32.8) (Fig. S1 Panel B). From raccoon dogs, one out of seven was positive (14.3 %, 95 % CI: 2.6–51.3), from pine marten, three out of six (50.0 %, 95 % CI: 10.0–90.0), while the sample from the stone marten and the other marten were negative. Antibodies to 16 different serogroups were detected in the 202 serum samples, while up to 5 different serogroups could be identified in a single sample (Table S1). The most prevalent serogroup in foxes was Javanica while in raccoons it was Javanica and Icterohaemorrhagiae (Fig. 3).

Out of the 21 PCR-positive animals, 4 were serologically negative. On the other hand, from the other 150 combined samples, the PCR was negative, but 35 were sero-positive.

All 166 kidney and urine samples tested PCR-negative for *Brucella* spp. as well as *F. tularensis*.

## 4 Discussion

This is the first study looking at *Leptospira* spp. sero- and PCR-prevalence in various wild carnivores focusing on foxes and raccoons in North-Eastern Germany. Overall PCR-prevalence was lower than sero-prevalence which might be because the bacteria have been cleared by the host or their number was too low to be detected, while antibodies indicate a historical infection. On the other hand, the few cases showing a sero-negative but PCR-positive result can be explained by a recent infection in which no antibody response has yet been mounted or by infections with unknown serogroups or serogroups not covered by the MAT.

Sero-prevalence in foxes was high with 34 % in our study using 19 serogroups but similar to Italy where 22.7 % was reported using 8 serogroups [17] or Poland with 26.3 % using 12 serogroups [18]. PCR-prevalence in foxes was lower with 11 % while in Italy 21 % were observed in a recent study [19].

While foxes belong to the native domestic fauna, raccoons have only recently been introduced to Germany and are considered “invasive alien species”. The raccoon population in North-Eastern Germany can be traced back to 1945 when several individuals escaped from a fur farm in Wolfshagen (Brandenburg) [7,20]. A second major point of raccoon introduction was determined in South-Western Germany where a recent study showed a PCR-prevalence of *Leptospira* spp. in raccoons of 3.9 % [21] which is in a similar range as the one of 1.3 % reported in Western Germany in a PhD thesis [22]. These values are much lower than the one observed in our study with 13.5 % as well as in a PhD study focusing on the same region and reporting a PCR-prevalence of 12.3 % [23]. Therefore, the prevalence of *Leptospira* spp. in raccoons could be different in the two different populations established in Western and Eastern Germany. Interestingly the PCR-prevalence was highest in raccoons and contrary to foxes, not much lower than sero-prevalence.

Successful genotype determination directly from clinical samples using the same MLST scheme has previously been achieved [24]. Our Sanger sequence data generated in this study do not indicate mixed infections. A high load of bacterial DNA needs to be present reflected by low Ct-values. Similar to our experience with *Mycoplasma hyopneumoniae*, a Ct-value below 30 is normally necessary for successful typing [25] which could be achieved in this study with four samples. In particular, ST17 identified in 50 % seems to be widely distributed in wild animals [19] and based on the pubMLST dataset (Fig. 3) is also highly associated with human cases. Given the advancement of DNA sequencing the MLST approach might replace the tedious MAT technique in the future, even though they have different indications.

The sample size for the other wild carnivores investigated was rather low and certainly more data should be gathered. Nevertheless, results corroborate the conclusion that similar to foxes and raccoons such animals can harbor *Leptospira* spp. and have to be regarded as a reservoir and a potential source for zoonotic infections. The risk of pets in urban and peri-urban areas or livestock on farmland acquiring leptospirosis should also be considered, especially in urban regions where foxes and raccoons can also permanently inhabit. The vaccination of dogs should therefore be promoted to mitigate the danger of infection in companion animals. The absence of *Brucella* spp. And *F. tularensis* points to a less critical situation regarding these two zoonotic pathogens, concluding that the risk of infection is therefore much lower, but again more specimens should be investigated especially with respect to raccoon dogs and martens to get a better idea of the prevalence of these zoonotic pathogens.

## Author contribution statement

All authors listed have significantly contributed to the development and the writing of this article.

## Funding statement

This study was supported by the research fund of the Institute of Veterinary Bacteriology, 10.13039/100009068University of Bern and the Multidisciplinary Center for Infectious Diseases, 10.13039/100009068University of Bern (Grant MCID_BPBB).

## Data availability statement

All data is included in the article.

## CRediT authorship contribution statement

**Peter Kuhnert:** Conceptualization, Formal analysis, Writing - original draft, Writing - review & editing. **Isabelle Brodard:** Investigation, Methodology. **Stefanie Ackermann:** Investigation, Methodology. **Peter Schierack:** Writing - review & editing, Resources. **Joerg Jores:** Conceptualization, Formal analysis, Funding acquisition, Project administration, Writing - original draft, Writing - review & editing.

## Declaration of competing interest

The authors declare that they have no known competing financial interests or personal relationships that could have appeared to influence the work reported in this paper.

## Figures and Tables

**figs1:**